# Dietary effects of *Garcinia kola* seed meal on growth performance, hematology and serum biochemical parameters of weaned rabbits

**DOI:** 10.14202/vetworld.2021.499-507

**Published:** 2021-02-25

**Authors:** Stanley Uzochukwu Ilo, Eunice Amaka Akuru, Jonathan Chinonso Egbo, Chika Ethelbert Oyeagu, Henry Oyeji Edeh

**Affiliations:** 1 Department of Animal Science, University of Nigeria Nsukka 410001, Nigeria; 2 Department of Livestock and Pasture Science, University of Fort Hare, Private Bag X1314, Alice 5700, Eastern Cape, South Africa; 3 Department of Agriculture, Faculty of Applied Sciences, Cape Peninsula University of Technology, Wellington Campus, Private Bag X8, Wellington 7654, Western Cape, South Africa

**Keywords:** *Garcinia kola*, performance, production, rabbits, supplementation

## Abstract

**Background and Aim:**

*Garcinia kola* seed (GKS) is used to prevent and cure a number of gastric-related ailments. GKS contain a copious amount of polyphenols and can be utilized as a natural growth promoter in the nutrition of weaned rabbits. This study aimed to determine the dietary effects of GKS meal (GKSM) on the growth performance, hematology and serum biochemical parameters of weaned rabbits.

**Materials and Methods:**

GKS were dried and ground into powder. Thirty-two 8-week-old crossbred rabbits with an average weight of 614 g were randomly divided into four dietary groups. The diets were designated as follows: Control (corn-soybean based diet with 0% GKSM) and the GKSM-supplemented diets with 1.5% GKSM, 3% GKSM, and 4.5% GKSM. On the 56^th^ day of the feeding trial, blood was collected from the marginal ear vein of all rabbits and transferred into two separate labeled tubes. The first set of blood was used to determine the hematological indices. The second set of blood was transferred into plain bottles and allowed to coagulate. The coagulated blood was subjected to standard methods of serum separation, and the sera were harvested and used to evaluate serum biochemical parameters.

**Results:**

Although the average final body weight was highest in rabbits fed with 1.5% GKSM; this value was similar to rabbits fed with 0% and 4.5% GKSM. The average daily weight gain was highest in rabbits fed with 1.5% GKSM, while the feed conversion ratio was improved in the 0% and 1.5% GKSM groups. The dietary treatments also had a significant effect on the red blood cell count (RBC) and hemoglobin (Hb) concentration, while other blood parameters did not differ significantly (p > 0.05). Higher inclusion levels (3% and 4.5%) of GKSM led to a significant increase in RBC and Hb values (p < 0.05). The total protein increased at all levels of GKSM inclusion (p < 0.05). Bilirubin, sodium, and potassium levels significantly decreased at 4.5% GKSM inclusion (p < 0.05). Urea levels were lowered at 1.5% and 4.5% GKSM inclusion, while cholesterol levels were decreased at 3% and 4.5% dietary levels.

**Conclusion:**

From the results of the present study, the supplementation of up to 4.5% GKSM revealed no harmful effect on the hematological and serum biochemical parameters of weaned rabbits, while their growth performance improved at a 1.5% inclusion level of GKSM.

## Introduction

Rabbit production is regarded as an important source of protein, income, food security, and employment generation in sub-Saharan African countries, including Nigeria [1,2]. With the increase in the human population, most African countries are battling the growing concern about the supply deficit in meat and other animal products [3]. In Nigeria, many of the available crop and animal protein sources are usually not sufficiently produced to meet the minimum daily protein needs of an average adult [3]. Hence, the reliance on rabbit production as a source of cheap animal protein in solving malnutrition cannot be overemphasized. Rabbits have a high reproduction rate, early maturing ability, rapid growth rate, and efficient feed utilization and can produce meat of high nutritional value [4]. From a nutritional and health standpoint, rabbit meat is suitable for all consumers [5]. This preference and attractiveness of rabbit meat over other animal meat sources may be due to its high amount of polyunsaturated fatty acids, low calorie, sodium, cholesterol, and fat contents [6,7]. Rabbit meat is a rich source of proteins, essential amino acids, B-vitamins, and minerals. It is highly digestible, wholesome, tasty, and contains higher energy that is mostly attributed to proteins. It also contains a low amount of purines with no uric acid [7,8]. There is an increased dependence on medicinal herbs as viable treatment options for several ailments by a large proportion of the worlds’ population, including 80% of those living in Africa [9]. Therefore, it has become necessary to investigate the possibilities of incorporating parts of plants in livestock and poultry feeds that will serve as growth enhancers and prophylactic agents [10,11]. Plant-derived medicines are relatively safe, accessible, and affordable, particularly to low-income earners in Africa [12]. Bitter kola (*Garcinia kola Heckel*) is among the spectrum of medicinal plants considered as traditional herbs in the health-care management of livestock [13,14].

*G. kola* is a medium-large tree of the *Clusiaceae* family that is commonly grown in West and Central Africa [10], which plays a key role in the African ethnomedicine and traditional ceremonies [15]. Apart from being one of the most traded non-timber forest products in these two regions of Africa, *Garcinia* kola seed (GKS) is a popular masticatory stimulant used by rural and urban dwellers to avoid and cure gastric related problems or simply for its distinctive astringent taste. GKS has received increased attention in recent times due to its rich content of flavonoids and other essential phenols. GKS contains an important compound known as kolaviron bioflavonoid [16]. Due to its bioflavonoid content, GKS has been reported to possess antimicrobial, antibacterial, antioxidant, anti-hepatoxic, hypoglycemic, hepatoprotective, growth-enhancing, radical scavenging, and aphrodisiac properties [17-20]. Arogba [21] reported that GKS contains 65% nitrogen-free extract (NFE) (carbohydrate), 70% moisture, 3.5%, crude protein (CP), 6.2% ether extract (EE), 1.5% ash, and 9.4% crude fiber (CF). GKS is a good source of essential fatty acids, such as linoleic (36 mg/kg) and oleic (38 mg/kg) acids, and amino acids, including leucine (1.9 g/kg), lysine (2.4 g/kg), and valine (1.7 g/kg) [22]. It also contains high amounts of Vitamin C [23] and minerals, such as potassium (25-722 mg/kg) and phosphorus (3.3-720 mg/kg) [24]. GKS contains low amounts of anti-nutritional factors, such as phytate, tannins, and oxalate, indicating a non-toxic consumption with no detrimental effect on humans and animals [23]. The low levels of oxalate in GKS show that it does not impair the absorption of important minerals (iron, magnesium, calcium, potassium, etc.) and the enzymatic digestion of proteins [25].

On the other hand, hematological and biochemical parameters are important diagnostic tools that are used to assess the physiological status of animals [26]. There are existing reports on feeding GKS meal (GKSM) to rats [19], fish [27,28], broilers [29,30], and layers [31]. However, there are a few studies to ascertain the dietary effects of GKSM on growth, hematology, and serum biochemical parameters of weaned rabbits. Therefore, this study was conducted to determine the growth performance and biomarker indices of weaned rabbits fed with different dietary levels of GKSM.

## Materials and Methods

### Ethical approval

All experimental procedures in the present study were performed according to the guidelines for the use of animals in biomedical research as approved and prescribed by the ethical research committee of the University of Nigeria, Nsukka.

### Study period and location

The experiment lasted for 56 days and was conducted in August and September, 2018 at the Rabbit Unit of the Department of Animal Science Teaching and Research Farm, University of Nigeria Nsukka (UNN), Nigeria. UNN lies at a latitude of 6.25^0^N and a longitude of 7.24^0^E.

### Procurement and processing of GKS

GKS were procured from the Ogige market in Nsukka, Enugu State, Nigeria. Processing of the seeds was done as previously described by Uko *et al*. [32] and Iwuji and Herbert [33]. The seeds were chopped into smaller pieces, air-dried, and milled into fine particles of 2 mm in diameter using a hammer mill.

### Proximate and phytochemical analysis

The methods described by the Association of Official Analytical Chemists [34] was used to determine the proximate contents of CP, CF, EE, ash, and NFE in GKSM (Table-1). A moisture-free 2.0 g of GKSM sample was weighed, placed in a crucible, and oven-dried for 24 h at 1000°C. The dry samples were removed from the oven, allowed to cool, weighed, and the dry matter (DM) was then determined. For ash determination, 10 g of the sample was incinerated in a muffle furnace for 6 h at 550°C. A 2.0 g of the sample was used for the CP analysis based on the Kjeldahl method. To determine the CP in the sample, the results were expressed in % nitrogen (N), which was multiplied with the conversion factor of 6.25 (N×6.25). The CF of the sample was determined based on the gravimetric method, 2 g of the sample was digested in a diluted sulfuric acid and sodium hydroxide, and the residue was then incinerated in a muffle furnace that was maintained for 5 h at 550°C. EE was extracted through the Soxhlet apparatus using petroleum ether as the extractant. The carbohydrate was obtained by the difference and was expressed as NFE. The flavonoid, saponins, and alkaloid contents of GKSM were determined using the gravimetric method described by Harborne [35]. The Folin–Dennis Spectrophotometric method described by Kirk and Sawyer [36] was used to determine the tannin content of the samples. Hydrogen cyanide determination was done using the method described by Onwuka [37]. Tables-1 and 2 show the proximate and phytochemical composition of GKSM.

**Table-1:** Proximate composition of *Garcinia kola* seed meal.

Parameters	Quantity
DM %	91.80
Ash %	0.95
EE %	0.90
Fiber %	3.65
Protein %	3.66
Carbohydrate %	82.64

EE=Ether extract, DM=Dry matter

**Table-2:** Phytochemical composition of *Garcinia* kola seed meal.

Parameters	Quantity (%)
Flavonoid	8.10
Alkaloids	8.20
Saponins	3.20
Tannins	0.02
Cyanide (mg/100)	0.64

### Experimental diets

Four experimental diets that were isocaloric and isonitrogenous were formulated to meet the rabbits’ dietary nutrient requirements. The diets were designated as follows:

1. Control diet: Maize-soybean-based diet with 0% additives
2. Control diet + 1.5% GKSM
3. Control diet + 3% GKSM, and
4. Control diet + 4.5% GKSM.

The nutrients and ingredients composition of the experimental diets is shown in Table-3.

**Table-3:** Ingredients and calculated nutrient composition of experimental diets.

Ingredients	T_1_(0%)	T_2_(1.5%)	T_3_(3.0%)	T_4_(4.5%)
Maize	45.00	44.00	43.00	42.50
Wheat offal	40.00	39.50	39.00	38.00
*Garcinia kola*	0.00	1.50	3.00	4.50
Fish meal	2.00	2.00	2.00	2.00
Blood meal	7.00	7.00	7.00	7.00
Soybean meal	4.00	4.00	4.00	4.00
Bone meal	0.25	0.25	0.25	0.25
Lysine	0.25	0.25	0.25	0.25
Methionine	0.25	0.25	0.25	0.25
Salt	0.25	0.25	0.25	0.25
Vit/min premix	1.00	1.00	1.00	1.00
Calculated composition %				
CP	17.21	17.09	16.98	16.85
Crude fibre	4.68	4.67	4.65	4.62
ME (kcal/kg)	3822	3789	3757	3725

*Supplied: Vit A: 10,000,000 IU; Vit. D3: 20,00,000 IU; Vit. E: 23,000 mg; Vit. K3: 2000 mg, Vit. B1: 1800 mg; Vit. B2: 5500 mg; Vit. B6: 3000 mg; Vit. B12: 15 mg; Niacin: 27,500 mg: Pantothenic acid: 7500 mg; Folic acid: 750 mg; Biotin: 60 mg; Chlorine: 300,000 mg; Co: 200 mg, Mn: 40,000 mg; Fe: 20,000 mg; Zn: 30,000 mg; I: 1000 mg; Cu: 3000 mg; Se: 200 mg; Antioxidant: 1250 mg. CP=Crude protein

### Management of experimental animals

Thirty-two 8-week-old crossbred rabbits with an average weight of 614 g were randomly selected and assigned to four experimental treatment groups of eight rabbits, each in a completely randomized design (CRD). The rabbits were housed in standard galvanized iron cages. Diets and clean water were given to the rabbits on an *ad libitum* basis throughout the feeding trial period.

### Evaluation of growth performance traits

At the beginning of the feeding trial, each rabbit was weighed to generate the initial weight of the animal before assigning them to different experimental pens. Thereafter, the rabbits were weighed weekly until the 56^th^ day (last day) of the feeding trial. The average daily weight was calculated using weekly weight readings. To calculate the average daily feed intake (ADFI), the daily feed refusal in the feeding trough was subtracted from the feed offered. The average daily weight gain (ADWG) and feed intake values were used to determine the feed conversion ratio (FCR) of the rabbits.

### Evaluation of hematology and serum biochemical parameters

Before blood collection, the rabbits were feed-fasted for 4 h in an attempt to allow the stabilization of various plasma constituents. Blood was collected in the morning to further reduce the variability of the measured plasma constituents. On day 56 of the feeding trial, 6 mL of blood was collected from the marginal ear vein of all rabbits using a sterile syringe and needles for the hematological and serum biochemical analysis. Blood collected was emptied into two separate labeled treated bottles. The first 3 mL of the blood was transferred into Bijou bottles containing ethylene diamine tetra-acetic acid and used for the determination of hematological parameters, such as packed cell volume (PCV), hemoglobin (Hb), red blood cell (RBC), white blood cell (WBC), and differentials leucocyte count, based on standard procedures [38]. The second 3 mL of the blood was transferred into plain bottles and allowed to coagulate. The coagulated blood was subjected to standard methods of serum separation, and the sera were harvested and used for evaluation of serum biochemical parameters. The total protein (TP), urea, cholesterol, bilirubin, and creatinine were determined using the Quimica Clinica Aplicada test kits (Quimica Clinica Aplicada, Spain) and a Spectrum lab 21A Spectrophotometer (Spectrum lab, England), following the manufacturers’ procedures [39,40].

### Statistical analysis

Data generated on performance, hematological and serum biochemical parameters were analyzed using the analysis of variance appropriate for CRD. Separation of means was done using the Duncan multiple range tests, and the statistical model used was described as follows: Y_ij_ = µ + T_i_+ E_ij_, where; µ= overall mean effect, T_i_= effect of the dietary treatment, and E_ij_ = Residual error associated with the observation of ij.

## Results

### Proximate and phytochemical analysis

The results on the proximate composition of GKSM used in this study (Table-1) showed that GKSM contains low moisture (8.2%) and high carbohydrate (82.64%) contents but had low amounts of CP (3.66%), CF (3.65), ash (0.95%), and EE (0.90%). The result of the phytochemical composition of GKSM in this study is presented in Table-2. The result showed that GKSM contained significant amounts of flavonoid (8.10%), alkaloids (8.20%) and saponin (3.20%), and small amounts of tannin (0.019%), and cyanide (0.64 mg/100 g).

### Performance characteristics

As shown in Table-4, the dietary supplementation of GKSM had a significant (p<0.05) effect on the average final body weight (AFBW), ADWG, and FCR, while the ADFI did not differ (p>0.05). Rabbits fed with 1.5% GKSM had the highest (p<0.05) AFBW, though their AFBW value was similar to those that received 0% (control) and 4.5% GKSM. The lowest AFBW was recorded in rabbits fed with 3% GKSM. ADWG was highest in rabbits fed with 1.5% GKSM and lowest in those that received 3% GKSM diets. Rabbits fed with a 1.5% GKSM diet had an improved FCR than the other treatments and similar to the control (0% GKSM diet).

**Table-4:** Performance characteristics of rabbits fed dietary *Garcinia* kola seed meal.

Parameters	T_1_(0%)	T_2_(1.5%)	T_3_(3%)	T_4_(4.5%)	SEM	p value
Av. initial body weight (g)	615.00	616.50	616.50	614.00	3.17	0.98
Av. final body weight (g)	1193.50^ab^	1224.50^a^	1041.50^b^	1145.00^ab^	28.65	0.05
Av. daily BWG (g)	10.33^b^	10.85^a^	7.59^d^	9.48^c^	2.85	0.01
Av. daily feed intake (g)	78.31	89.17	73.58	90.66	3.21	1.61
FCR (g/g)	7.58^bc^	8.22^b^	9.69^a^	9.56^a^	0.15	0.01

a,b,c Means within columns with different superscripts differ significantly (p<0.05). PCV=Packed cell volume, Av=Average, BWG=Body weight gain, SEM=Standard error of mean, FCR=Feed conversion ratio

### Hematological indices

Table-5 represents the hematological traits of rabbits fed with different inclusion levels of GKSM. The dietary treatments significantly (p<0.05) influenced the RBC count and Hb concentration, while the PCV, WBC count, lymphocyte, neutrophils, monocytes, basophils, and eosinophil did not differ (p>0.05). The RBC value was highest (p>0.05) in rabbits fed with 4.5% GKSM, statistically similar with rabbits that fed 1.5% and 3% GKSM diets, while the lowest RBC value was recorded in the control group. The highest (p<0.05) Hb value was recorded in rabbits fed with 3% and 4.5% GKSM diets, while the lowest value was seen in the control and 1.5% GKSM-treated rabbits.

**Table-5:** Hematological indices of rabbits fed varying dietary levels of *Garcinia* kola seed meal.

Parameters	T_1_(0%)	T_2_(1.5%)	T_3_(3.0%)	T_4_(4.5%)	SEM	p value
PCV (%)	26.50	26.50	26.00	27.00	0.59	0.98
WBC (x10^3^/mm^3^)	11.40	10.50	11.05	11.50	2.39	0.71
RBC (x10^12^/L)	9.43^b^	10.12^ab^	10.35^ab^	10.56^a^	0.52	0.04
Hb (g/dl)	10.50^b^	10.95^b^	11.80^a^	11.60^a^	0.22^a^	0.04
Lymphocyte (%)	74.00	78.00	73.50	81.00	1.80	0.50
Neutrophil (%)	25.00	20.00	24.50	18.50	1.70	0.54
Monocyte (%)	0.51	0.51	1.50	0.51	0.25	0.48
Basophil (%)	0.51	0.50	0.51	0.01	0.18	0.81
Eosinophil (%)	0.01	0.51	0.01	0.01	0.12	0.48

a,b,c Means within columns with different superscripts differ significantly (p<0.05). PCV=Packed cell volume, WBC=White blood cell count, RBC=Red blood cell count, Hb=Hemoglobin concentration, SEM=Standard error of mean

### Serum biochemical parameters

The serum biochemical parameters of rabbits fed with different inclusion levels of dietary GKSM are presented in Table-6. The supplementation of dietary GKSM influenced the TP, bilirubin, urea, cholesterol, sodium, and potassium contents (p<0.05). However, the concentrations of creatinine, albumin, calcium, and phosphorus did not differ (p>0.05). The TP concentration was highest in rabbits fed GKSM-supplemented diets (p<0.05), while the lowest concentration was seen in the control diet (p<0.05). The highest bilirubin contents were recorded in rabbits fed with the control diet (p<0.05), although statistically similar with those that received 1.5% and 3% inclusion levels of GKSM, while rabbits fed with 4.5% GKSM had the lowest value for bilirubin (p<0.05). Rabbits fed with 3% and 4.5% GKSM had the lowest values for cholesterol (p<0.05), while the highest cholesterol content was observed among the control group and the 1.5%-GKSM supplemental group (p<0.05). The highest urea content was recorded in rabbits fed on the control diet (p<0.05), statistically similar with those that received the 3%-GKSM diet. Rabbits fed with 1.5% and 4.5% GKSM diets had the lowest values for urea (p<0.05). Sodium (Na) and potassium (K) concentrations were lowest in rabbits fed with the 4.5%-GKSM diet than rabbits fed on other dietary treatments (p<0.05). The highest potassium values were seen in rabbits fed with 1.5% and 3% GKSM diets and those fed on the control diets (p<0.05), while the highest sodium concentration value was recorded in rabbits fed on the control diet (p<0.05).

**Table-6:** Serum biochemical indices of rabbits fed varying dietary levels of *Garcinia kola* seed meal.

Parameters	T_1_(0%)	T_2_(1.5%)	T_3_(3.0%)	T_4_(4.5%)	SEM	P value
Total protein (g/dl)	4.34^b^	6.39^a^	6.62^a^	6.98^a^	0.39	0.04
Bilirubin (mg/dl)	2.47^a^	2.44^ab^	2.43^ab^	2.38^b^	0.13	0.05
Urea (g/dl)	6.25^a^	4.55^b^	4.90^ab^	4.65^b^	0.30	0.05
Cholesterol (mg/dl)	74.00^a^	67.00^ab^	62.00^b^	60.00^b^	2.38	0.05
Creatinine (mg/dl)	2.47	2.39	2.70	3.47	0.26	0.54
Albumin (g/dl)	4.52	4.37	4.39	4.35	0.04	0.36
Sodium (mmol/l)	12.46^a^	11.70^b^	11.91^b^	10.42^c^	0.29	0.00
Potassium (mg/dl)	8.96^a^	7.38^a^	7.82^a^	4.65^b^	0.63	0.02
Calcium (mg/dl)	10.45	10.05	10.87	9.88	0.24	0.56
Phosphorus (mg/dl)	6.34	6.28	5.89	6.32	0.12	0.58

a,b,c Means within columns with different superscripts differ significantly (p<0.05), SEM; Standard error of mean

## Discussion

### Proximate and phytochemical analysis of GKSM

The present result on carbohydrates, i.e., NFE (Table-1) is in line with a previous report on the rich carbohydrate content of GKSM [41]. Carbohydrates serve as a ready and accessible energy source required for physical activity and nerve tissue regulation [42]. The values reported for other proximate contents, such as DM, CP, crude fat, ash, and crude, are similar to the values reported by Arogba [21] and Odebunmi *et al*. [24]. The low moisture content of 8.2% recorded for GKSM (Table-1) indicates its capacity for long-term storage with minimal microbial invasion. The phytochemical results (Table-2) of this study were within the results of Adesuyi *et al*. [41] for all the parameters measured. Phytochemicals are biologically active compounds, which, though they are not established nutrients, contribute significantly to protecting biological systems against degenerative diseases [43].

### Growth performance indices

Medicinal plants can enhance growth responses in animals due to their antibacterial, antioxidant, antimicrobial, and physiological properties [44,45] due to their inherent flavonoid and phenols content [46,47]. These bioactive compounds inherent in the medicinal plants give them the ability to decrease the population of growth-depressing gut microbial metabolites, thereby increasing the available nutrients for the animal’s utilization [48]. Flavonoids are non-nutritive plant components found commonly in different plant parts, such as flowers, barks, fruits, nuts, and seeds [28]. There are reports that GKSM promotes growth in animals due to its bioflavonoid contents [23]. Bioflavonoid compounds are known to contain estrogen, which enhances growth [49]. We observed that 1.5%-GKSM diet significantly improved the final body weight of rabbits with the better conversion of feed-to-meat ratio (Table-4), and they competed favorably with the control diet. The improved final body weight and better conversion of feed-to-meat ratio recorded in rabbits fed dietary 1.5% GKSM may be due to GKSM increasing nutrient absorption from the gastrointestinal tract [50] at this level (1.5% GKSM). This result agrees with the findings of Nyadjeu *et al*. [28], who reported that dietary GKSM significantly enhanced growth response in juvenile fish.

The ADWG was significantly improved at a 1.5% GKSM inclusion than the control diet (without GKSM). According to Alabi *et al*. [50], an increase in ADWG was reported in rats fed with kolaviron (bioflavonoid complex of GKSM) supplemental diets. Rabbits that received higher dietary levels (3% and 4.5%) of GKSM had reduced ADWG than the control (Table-4). Ebenebe *et al*. [51] reported that as the dietary inclusion levels of *G. kola* increased from 2.5% to 7.5%, the weight gain of the rabbits was significantly depressed. Higher levels of GKSM had been previously reported to have growth-depressing effects on rabbits [31,52] and broiler birds [30]. The growth-depressing effects of higher GKSM may be due to the presence of tannins and oxalate. These anti-nutrient factors (ANFs) reduce the bioavailability of minerals, such as calcium, which animals use. Higher tannins also bind to available proteins and make them either indigestible or unpalatable to animals [30], reducing the growth response. Interestingly, GKSM used in this study had a considerably low tannin content of 0.02% (Table-2). Omeh *et al*. [53] and Dah-Nouvlessounon *et al*. [54] also reported that GKSM contains low amounts of tannins, oxalate, phytate, and trypsin inhibitors. Hence, it is difficult to attribute the concentration of tannins to the low AFBW and ADWG observed at 3% and 4.5% levels of GKSM inclusion in this study. Nonetheless, Ebenebe *et al*. [51] reported that apart from the percentage inclusion level, the feeding duration of GKSM to animals may also contribute to its growth-depressing effect.

The FCR was improved at the 1.5% level of GKSM compared with 3% and 4.5% levels of GKSM. However, this FCR improvement did not translate into a better AFBW and ADWG for rabbits fed on a 1.5% GKSM diet (Table-4). Kaur and Shah [55] reported that a 3% inclusion of GKSM had a significant reduction in bile secretion and digestive enzyme activities, resulting in a noticeable decrease in ADWG of rabbits. Nonetheless, Mohammed and AbdulMalik [56] reported that the dietary supplementation of 5 g/kg GKSM significantly improved the FCR of broiler birds.

### Hematological indices

Blood indices are useful pointers to the biological effects of nutritional components on animals. Usually, any change in the physiological state of the animals suggests alterations in their hematological values [25,57]. In this study, we observed that the dietary inclusion of GKSM significantly increased the RBC and Hb (Table-5). RBCs contain Hb, which is responsible for oxygen transport to needy tissues. There is evidence that GKS has the potential to normalize abnormal hematological indices associated with diabetes [58]. According to Oluyemi *et al*. [59], *G. kola* has erythropoietic effects due to its ability to inhibit the destruction of RBC by reactive oxygen species (ROS). This ability to effectively scavenge ROS that destroys RBC and reduces lipid peroxidation in the membranous tissues of erythrocytes is due to the antioxidant capacity of GKS [58]. In the current study, the rabbits fed on the control diet had reduced RBC and Hb compared with the GKSM-treated rabbits. An earlier report [60] showed that an improvement in the values of circulating RBC in animals is suggestive of an increased ability to withstand respiratory stress. Similarly, Mohammed and Oloyede [61] linked the depletion of erythrocytes (RBCs) to anemia. Hence, the lower RBCs and Hb values recorded for the control rabbits may be due to the stress associated with anti-nutritional factors, which often cause depletion in the oxygen-carrying capacity of the animal’s blood, leading to an impaired growth response [61]. Our result is contrary to the findings of Ebenebe *et al*. [51], who reported a reduction in the Hb values among rabbits fed with GKS diets. The reports of Udenze *et al*. [58] also did not align with our current findings. They reported that the dietary supplementation of GKSM significantly decreased the RBC of diabetic rats. The hematological values reported in the present study are within the normal physiological range for growing rabbits [62].

### Serum biochemical parameters

The TP is made up of albumin and globulin, the sum of which is a pointer to the total amount of protein in the blood. TP is a key plasma constituent used to assess the health and functional status of some important organs (e.g., liver and kidney), and the nutritional status of animals. Elevated TP levels were observed at all dietary inclusion levels of GKSM compared with the control diet (Table-6). The result of the present study does not agree with the findings of Ebenebe *et al*. [9], who reported that the supplemental *G. kola* reduced plasma protein in rabbits. Esiegwu *et al*. [31] also reported that serum protein did not differ with the dietary supplementation of GKSM in rabbits. There is evidence that suggests that an increase in serum TP may be due to the nutritional effects of the dietary treatments and an improved body weight gain [63]. Krames [64] also affirmed that elevated TP levels may be due to enhanced protein synthesis, which is an indication of the normal functioning of the liver, and ultimately, better growth performance. In this study, an improved performance (final body weight, ADWG, and FCR) was only observed at a 1.5% GKSM supplementation (Table-4). Nevertheless, there are reports that an elevated TP may be associated with acute inflammatory response, dehydration, and certain kinds of tissue damage [65]. Although no histopathological examination of the gut was done in this study, the reduced urea levels recorded among the GKSM groups showed that GKSM did not exert any toxic effect on the rabbits.

The significant reduction of bilirubin levels at a 4.5% GKSM inclusion suggests that liver functions were not impaired. According to Alabi *et al*. [50], an elevation in the amount of serum bilirubin indicates bile duct obstruction and impairment in the secretory function of the liver (liver necrosis). The serum urea depends on the quality and quantity of dietary protein [66]. Higher levels of blood urea observed in the control group in this study may be due to the presence of ANFs, which may have reduced the protein quality and caused an imbalance in the amino acid composition of the diet [67]. An increase in the urea levels had also been linked to renal dysfunction [68]. Interestingly, at 1.5% and 4.5% inclusion levels, GKSM was able to significantly lower the blood urea concentration (Table-6), indicating that GKSM did not impair kidney function. Our findings are in agreement with the reports of Adaramoye [69]. According to the author, administering kolaviron at 100 mg/kg to streptozotocin-diabetic rats decreased their serum urea levels. The GKS and its bioflavonoid have protective effects against diabetic-induced renal dysfunction as a result of its antioxidant property [70]. The Na and K concentrations were lowest at a 4.5% GKSM inclusion than the other dietary treatments (Table-6). Increased serum Na and K levels had earlier been attributed to the inability of the kidney to properly regulate these electrolytes, indicating renal dysfunction [71]. Esiegwu *et al*. [31] reported that GKSM supplementation did not affect serum K and Na levels in rabbits.

The onset and progression of atherosclerosis have been linked to hyperlipidemia [72]. Atherosclerosis has been implicated as a major cause of several coronary heart disease conditions, such as myocardial infarction, stroke, and ischemic heart disease, which result in a huge number of deaths worldwide [73]. Medicinal plants with lipid-reducing properties have been shown as effective tools for reducing and preventing coronary heart diseases [74,75]. GKS has an ameliorative effect in hyperlipidemia due to its bioflavonoid content [76]. According to Adejor *et al*. [77], GKS can reduce total cholesterol concentrations due to its ability to decrease the activity of hepatic 3-hydroxyl-3-methyl glutaryl coenzyme A reductase, which is a rate-limiting enzyme involved in cholesterol biosynthesis. The authors also affirmed that the lipid-lowering effects of GKS are attributable to its ability to stimulate cholesterol-7-alpha-hydroxylase, which is responsible for the conversion of cholesterol into bile acids. We observed that GKSM used in this study exhibited high hypolipidemic activity at higher inclusion levels of 3% and 4.5% than the control and 1.5% dietary groups (Table-6). Our results do not align with the reports of Esiegwu *et al*. [31] who did not record any treatment effect on serum cholesterol on feeding rabbits with GKSM.

## Conclusion

From the results obtained in this study, it was observed that a 1.5% supplementation of GKSM significantly improved the average daily body weight and FCR of rabbits, while the concentrations of urea, bilirubin, and cholesterol were significantly decreased at 4.5% inclusion than the control diet. Thus, we concluded that supplementation of up to 4.5% of GKSM had no harmful effect on the hematological and serum biochemical parameters of weaned rabbits, while the growth performance of the animals was improved at a 1.5% inclusion level of GKSM.

## Authors’ Contributions

SUI: Designed and supervised the study, collected and analyzed data, drafted, and revised manuscript. EAA: Drafted and revised manuscript. JCE: Designed the study, collected data, revised manuscript. CEO: Analyzed data and revised manuscript. HOE: Revised manuscript. All authors read and approved the final manuscript.

## References

[1] Oseni S.O, Lukefahr S.D (2014). Rabbit production in low-input systems in Africa:Situation, knowledge, and perspectives a review. World Rabbit Sci.

[2] Tembachako D.S, Mrema M.N.J (2016). Factors affecting the production of rabbits by smallholder farmers in Mt Darwin District of Zimbabwe. Amity J Agribus.

[3] Schonfeldt H.C, Hall N.G (2012). Dietary protein quality and malnutrition in Africa. Br. J. Nutr.

[4] Szendro Z.S, Zotte A.D (2011). Effect of housing conditions on production and behavior of growing meat rabbits:A review. Asian Australas. J. Anim. Sci.

[5] Amaravadhi A.C, Mallam M, Manthani G.P, Komireddy K.R (2012). Effect of dietary supplementation of probiotics and enzymes on the haematology of rabbits reared under two housing systems. Vet. World.

[6] Zotte A.D (2014). Rabbit meat for farming purposes. Anim. Front.

[7] Zotte A.D, Szendro Z.S (2011). The role of rabbit meat as functional food:A review. Meat Sci.

[8] Cardinali R, Cullere M, Bosco A.D, Mugnai C, Ruggeri S, Mattioli S, Castellini C, Marinucci M.T, Zotte A.D (2015). Oregano, rosemary and Vitamin E dietary supplementation in growing rabbits:Effect on growth performance, carcass traits, bone development and meat chemical composition. Livest. Sci.

[9] Ebenebe C.I, Anizoba M.A, Onu C.O, Okeke J.J, Ufele A.N (2010). Effect of graded levels of Garlic (*Allium sativum*) and Ginger (*Zingiber officinale*) mixtures on the growth performance and microbial load of weaner rabbits. Int. J. Biol. Sci.

[10] Dranca F, Oroian M (2016). Optimization of ultrasound-assisted extraction of total monomeric anthocyanin (TMA) and total phenolic content (TPC) from eggplant (*Solanum melongena L.) peel*. Ultrason. Sonochem.

[11] Altemimi A, Lakhssassi N, Bharlouei A, Watson D, Lightfoot D (2017). Phytochemicals:Extraction, isolation, and identification of bioactive compounds from plant extracts. Plants (Basel).

[12] Zhang R, Zhou J, Jia Z, Zhang Y, Gu G (2004). Hypoglycemic effect of *Rehmannia glutinosa* oligosaccharide in hyperglycemic and alloxan-induced diabetic rats and its mechanism. J. Ethnoparmacol.

[13] Oboh G, Ogunsuyi O.B, Oyelade M.T, Akomolafe S.F (2018). Effect of dietary inclusions of bitter kola seed on geotactic behavior and oxidative stress markers on *Drosophilia melongaster*. Food Sci. Nutr.

[14] Obun C.O, Kehinde A.S, Osaguona P.O (2013). The Effects of Feeding Broiler Chicks Graded Levels of Sun-cured Neem (*Azadirchta indica* A Juss.) Leaf Meal on Blood Chemistry. Proceeding 38^th^ Annual Conference NIG, Society of Animal Production, River State, Nigeria.

[15] Manourova A, Leuner O, Tchoundjeu Z, Van Damme O.P.V, Verner V, Pribyl O, Lojka B (2019). Medicinal potential, utilization and domestication status of bitter kola (*Garciniakola hecke* L) in West and Central Africa. Forests.

[16] Konziase B (2015). Protective activity of bioflavonones from *Garcinia kola* against *Plasmodium* infection. J. Ethnopharmacol.

[17] Kanmegne G, Mboudbda H.D, Temfack B, Koffi E.K, Omokolo D.N (2010). Impact of biochemical and morphological variations on germination traits in *Garcinia kola* Heckel seeds collected from Cameroon. Res. J. Seed Sci.

[18] Usunomena U (2012). Review manuscript:A review of some African medicinal plants. Int. J. Pharm. Bio Sci.

[19] Yakubu M.T, Quadri A.L (2012). *Garcinia kola* seeds:Is the aqueous extract a true aphrodisiac in male Wistar rats?. Afr. J. Tradit. Complement. Altern. Med.

[20] Oyenihi O.R, Brooks N.L, Oguntibeju O.O (2015). Effect of kolaviron on hepatic oxidative stress in streptozotocin-induced diabetes. BMC Complement. Altern. Med.

[21] Arogba S.S (2000). Comparative analyses of the moisture isotherms, proximate composition, physical and functional properties of dried *Cola nitida* and *Garcinia kola* kernels. J. Food Compos. Anal.

[22] Elenyimi A.F, Bressler D.C, Amoo I.A, Sporns P, Oshodi A.A (2006). Chemical composition of bitter cola (*Garcinia kola*) seed and hulls. Pol. J. Food Nutr.

[23] Onyekwelu J.C, Mosandl R, Oyewale O, Stimm B (2015). Antioxidant, nutritional and anti-nutritional composition of *Garcinia kola* and *Chrosophyllumalbidum* from rainforest ecosystem of Ondo State, Nigeria. J. Forest. Res.

[24] Odebunmi E.O, Oluwaniyi O.O, Awoola G.V, Adediji O.D (2009). Proximate and nutritional composition of kola nut (*Cola nitida*), bitter cola (*Garcinia kola*) and alligator pepper (*Afromomum melegueta*). Afr. J. Biotechnol.

[25] Abidemi T.A, Adebayo O.J, Idowu O, Agbotoba M.O (2009). Nutrient content and anti-nutritional factors in shea butter (*Butryospermum parkii*) leaves. Afr. J. Biotechnol.

[26] Olabanji R.O, Oyebiyi O.O, Tona G.O, Olagun O (2009). Haematological and Serum Biochemical Response of Growing Rabbits Fed Diets Containing Processed Mango (*Mangifera indica*) Seed Kernel Meal. Proceeding 14^th^ Annual Conference.

[27] Dada A.A, Ikeurowo M (2009). Effects of ethanolic extracts of *Garcinia kola* seeds on growth and haematology of catfish (*Clarias gariepinus*) broodstock. Afr. J Agric. Res.

[28] Nyadjeu P, Angoun J, Ndasi N.P, Tabi-Tomedi M.E (2019). Effect of *Garcinia kola* seeds supplemented diet on growth performance and gonadal development of *Oreochromis niloticus* juveniles breed in ponds. Fish. Aquat. Sci.

[29] Esiegwu A.C, Udedibie A.B.I (2009). Growth performance of and antimicrobial activities in broilers fed supplementary bitter kola (*Garcinia kola*). Anim. Prod. Res. Adv.

[30] Ibekwe H.A, Eteng M.U, Meremikwu V.N (2010). Evaluation of growth performance and haematological response of broiler chicks to raw and boiled *Garcinia kola* seed diet. Niger. Vet. J.

[31] Esiegwu A.C, Enyenihi G.E, Emanalom O.O, Okoli I.C, Udedibie A.B.I (2013). Effect of graded dietary levels of *Garcinia kola* seed meal on performance, intestinal microbial load, haematological and serum biochemical profile of rabbits. Agro-Science.

[32] Uko O.J, Usman A, Mohammed A (2001). Some biological activities of *Garcinia kola* in growing rats. Vet. Archiv.

[33] Iwuji T.C, Herbert U (2015). Haematological and serum biochemical characteristic of rabbit bucks fed diets containing *Garcinia kola* seed meal. J. Nat. Sci. Res.

[34] Association of Official Analytical Chemists (1990). Association of Official Analytical Chemist, Official Methods of Analysis, Method 930.05.

[35] Harbone J.B (1973). Phytochemical Methods. Guide to Modern Techniques of Plant Analysis.

[36] Kirk R.S, Sawyer R (1998). Pearson's Composition and Analysis of Food.

[37] Onwuka G.C (2005). Food Analysis and Instrumentation:Theory and Practice.

[38] Jain N.C (1993). Essential of Veterinary Hematology.

[39] Meyer D.J, Coles E.H, Rich L.J (1992). Veterinary Laboratory Medicine Interpretation and Diagnosis.

[40] Evans G.O (1996). Animal Clinical Chemistry:A Primer for Toxicologist.

[41] Adesuyi A.O, Elumm I.K, Adaramola F.B, Nwokocha A.G.M (2012). Nutritional and phytochemical screening of *Garcinia kola*. Adv. J. Food Sci. Technol.

[42] Whitney E.N, Rolfes S.R (2005). Understanding Nutrition.

[43] Dreosti I.E (2000). Recommended dietary intake levels for phytochemicals:Feasible or Fanciful?. Asia Pac. J. Clin. Nutr.

[44] Hosseinzadeh H, Qotbi A.A.A, Seidavi A, Norris D, Brown D (2014). Effects of different levels of coriander (*Coriandrum sativum*) seed powder and extract on serum biochemical parameters, microbiota and immunity in broiler chicks. Sci. World J.

[45] Abdelnour S, Alagawany M, Abd El-Hack M, Sheiha A, Swelum A, Saadeldin I (2018). Growth, carcass traits, blood haematology, serum metabolites, immunity and oxidative indices of growing rabbit fed diets supplemented with red or black pepper oils. Animals (Basel).

[46] Alagawany M, Abd El-Hack M, Al-Sagheer A, Naiel M, Saadeldin I, Swelum A (2018). Dietary cold pressed watercress and coconut oil mixture enhances growth performance, intestinal microbiota, antioxidant status and immunity of growing rabbits. Animals (Basel).

[47] Abd El-Hack M.E, Mahgoub S.A, Hussein M.M.A, Saadeldin I.M (2018). Improving growth performance and health status on meat-type quail by supplementing the diet with black cumin cold-pressed oil as a natural alternative for antibiotics. Environ. Sci. Pollut. Res.

[48] Mountzouris K.C, Paraskevas V, Tsirtsikos P, Palamidi I, Steiner T, Schatzmayr G, Fegeros K (2011). Assessment of a phytogenic feed additive effect on broiler growth performance, nutrient digestibility and caecal microflora composition. Anim. Feed Sci. Technol.

[49] Kocour M, Lynhard O, Gela D, Rodina M (2005). Growth performance of all female and mixed-sex common carp *Cyprinus carpio* L population in central European climatic conditions. J. World Aquac. Soc.

[50] Alabi Q.K, Akomolafe R.O, Olukiran O.S, Nafiu A.O, Omole J.G, Adefisayo A.M, Oladele A.A (2017). Assessment of haematological and biochemical effects of kolaviron in male Wistar rats. Br. J. Pharm. Res.

[51] Ebenebe C.I, Nwankwor A.O, Izukanne R.O, Ufele A.N (2016). Performance and haematological parameters of rabbits fed graded levels of bitter kola (*Garcinia kola*). Int. J. Livest. Prod.

[52] Obi A.U, Nwoha P.U (2014). Effects of kolaviron, the major constituents of *Garcinia kola* on the histology of the hypothalamus, pituitary and testes using adult male Wistar rats a model organism. Forensic Med. Anat. Res.

[53] Omeh Y.N, Onoja S.O, Ezeja M.I, Uchendu W.C, Okorie E, Raymond M (2014). Quantitative phytochemical, proximate analysis and hypolipidemic effect of *Garcinia kola*. Br. J. Med. Res.

[54] Dah-Nouvlessounon D, Adjanohoun A, Sina H, Noumavo P.A, Diarrasouba N, Parkouda C, Madode Y.E, Dicko M.H, Baba-Moussa L (2015). Nutritional and anti-nutrient composition of three kola nuts (*Cola nitida*, *Cola acuminata* and *Garcinia kola*) produced in Benin. Food Nutr. Sci.

[55] Kaur R, Shah T.K (2017). A review on role of plant waste products on fish growth, health and production. J. Entomol. Zool. Stud.

[56] Mohammed A.A, AbdulMalik M.A (2013). Effect of bitter kola (*Garcinia kola*) as dietary additive on the performance of broiler chicks. J. Environ. Ecol.

[57] Toghyani M, Toghyani M, Gheisari A.A, Ghalamkari G, Eghbalsaeid S (2011). Evaluation of cinnamon and garlic as antibiotic growth promoter substitutions on performance, immune responses, serum biochemical and haematological parameters. Livest. Sci.

[58] Udenze E.C, Ezirim A.U, Ihedimbu C.P, Iheme C.I (2014). Effect of oral administration of *Garcinia kola* seeds on haematological and defence parameters of diabetic rats. Am. J. Biochem. Mol. Biol.

[59] Oluyemi K.A, Jimoh O.R, Adesanya O.A, Omotuyi I.O, Josiah S.J, Oyesola T.O (2007). Effects of crude ethanolic extract of *Garcinia cambogia* on the reproductive system of Wistar rats (*Rattus novergicus*). Afr. J. Biotechnol.

[60] Afolabi K.D, Akinsoyinu A.O, Abdullah A.R.O, Olajide R, Akinleye S.B (2011). Haematological parameters of the Nigerian local grower chickens fed varying dietary levels of palm kernel cake. Poljoprivreda.

[61] Mohammed N.O, Oloyede O.B (2009). Growth performance of broiler chicks fed *Aspergillus niger*-fermented *Terminalia catappa* seed meal based diet. Glob. J. Biotechnol. Biochem.

[62] Research Animal Resource (2009). Reference Values for Laboratory Animals. Normal Haematological Values.

[63] Kapelanski W, Grajewska S, Bocian M, Dybala J, Jankowiak H, Wisniewska J (2004). Changes in blood biochemical indicators during fattening of the high-lean pigs. Anim. Sci. Pap. Rep.

[64] Krames (2010). Total protein and A/G ratio tests. Mount Nittany Med. Center.

[65] Murray R.K, Bender D.A, Botham K.M, Kennelly P.J, Rodwell V.W, Weil P.A (2012). Harper's Illustrated Biochemistry.

[66] Iyayi E.A, Tewe O.O (1998). Serum total protein, urea and creatinine levels as indices of quality of cassava diet for pigs. Trop. Vet.

[67] Fanimo A.O, Oduguwa O.O, Onifade A.O, Olutunde T.O (2000). Protein quality of shrimp-waste meal. Bioresour. Technol.

[68] Seki M, Nakayama M, Sakoh T, Yoshitomi R, Fukui A, Katafuchi E, Tsuda S, Nakano T, Tsuraya K, Kitazono T (2019). Blood urea nitrogen is independently associated with renal outcomes in Japanese patients with stage 3-5 chronic kidney disease: A prospective observational study. BMC Nephrol.

[69] Adaramoye O.A (2012). Antidiabetic effect of kolaviron, a bioflavonoid complex isolated from *Garcinia kola* seeds, in Wistar rats. Afr. Health Sci.

[70] Iwuji T.C, Herbert U (2012). Evaluation of the growth-promoting effect of *Garcinia kola* seed in growing rabbits. J. Glob. Biosci.

[71] Oboh H.A, Olumese F.E (2010). Effects of low-carbohydrate fat, Nigerian-like diet on biochemical indices in rabbits. Pak. J. Nutr.

[72] Hannawi S, Hannawi H, Al-Salmi I (2020). Cardiovascular disease and subclinical atherosclerosis on rheumatoid arthritis. Hypertens. Res.

[73] Vaziri N.D, Norris K (2011). Lipid disorders and their relevance to outcomes in chronic kidney disease. Blood Purif.

[74] Geetha G, Kalavalarasariel G.P, Sankar V (2011). Antidiabetic effect of *Achyranthes rubrofusca* leaf extracts on alloxan-induced diabetic rats. Pak. J. Pharm. Sci.

[75] Shah S.M.A, Akram M, Riaz M, Muniri N, Rasool G (2019). Cardioprotective potential of plant-derived molecules: A scientific and medicinal approach. Dose Response.

[76] Patel D.K, Patel K.A, Patel U.K, Thounaojam M.C, Jadeja R.N, Ansarullah P.G.S, Salunke S.P, Devkar R.V, Ramachandran A.V (2009). Assessment of lipid lowering effect of *Sida rhomboidea*. Roxb methanolic extract in experimentally induced hyperlipidemia. J. Young Pharm.

[77] Adejor E.B, Ameh D.A, James D.B, Owolabi O.A, Ndidi U.S (2017). Effect of *Garcinia kola* bio flavonoid fractions on serum lipid profile and kidney function parameters in hyperlipidemic rats. Clin. Phytosci.

